# A Meta-Analysis of Unilateral versus Bilateral Pedicle Screw Fixation in Minimally Invasive Lumbar Interbody Fusion

**DOI:** 10.1371/journal.pone.0111979

**Published:** 2014-11-06

**Authors:** Zheng Liu, Qi Fei, Bingqiang Wang, Pengfei Lv, Cheng Chi, Yong Yang, Fan Zhao, Jisheng Lin, Zhao Ma

**Affiliations:** 1 Department of Orthopaedics, Peking University Shougang Hospital, Beijing, China; 2 Department of Orthopaedics, Beijing Friendship Hospital, Capital Medical University, Beijing, China; Toronto Western Hospital, Canada

## Abstract

**Study Design:**

Meta-analysis.

**Background:**

Bilateral pedicle screw fixation (PS) after lumbar interbody fusion is a widely accepted method of managing various spinal diseases. Recently, unilateral PS fixation has been reported as effective as bilateral PS fixation. This meta-analysis aimed to comparatively assess the efficacy and safety of unilateral PS fixation and bilateral PS fixation in the minimally invasive (MIS) lumbar interbody fusion for one-level degenerative lumbar spine disease.

**Methods:**

MEDLINE/PubMed, EMBASE, BIOSIS Previews, and Cochrane Library were searched through March 30, 2014. Randomized controlled trials (RCTs) and controlled clinical trials (CCTs) on unilateral versus bilateral PS fixation in MIS lumbar interbody fusion that met the inclusion criteria and the methodological quality standard were retrieved and reviewed. Data on participant characteristics, interventions, follow-up period, and outcomes were extracted from the included studies and analyzed by Review Manager 5.2.

**Results:**

Six studies (5 RCTs and 1 CCT) involving 298 patients were selected. There were no significant differences between unilateral and bilateral PS fixation procedures in fusion rate, complications, visual analogue score (VAS) for leg pain, VAS for back pain, Oswestry disability index (ODI). Both fixation procedures had similar length of hospital stay (MD = 0.38, 95% CI = −0.83 to 1.58; P = 0.54). In contrast, bilateral PS fixation was associated with significantly more intra-operative blood loss (P = 0.002) and significantly longer operation time (P = 0.02) as compared with unilateral PS fixation.

**Conclusions:**

Unilateral PS fixation appears as effective and safe as bilateral PS fixation in MIS lumbar interbody fusion but requires less operative time and causes less blood loss, thus offering a simple alternative approach for one-level lumbar degenerative disease.

## Introduction

Minimally invasive (MIS) lumbar interbody fusion is a popular technique used to treat various lumbar degenerative disorders. Over the traditional open surgery, MIS surgery offers multiple advantages; it not only reduces the approach-related muscle damage, blood loss, postoperative pain, length of hospital stay, and postoperative narcotic usage but also allows for early ambulation. Accordingly, MIS is popularized by most of the spine surgeons, especially when it is used with percutaneous pedicle screws [1].

Generally, bilateral pedicle screw (PS) fixation is accepted as a standard procedure in lumbar interbody fusion. Providing rigid fixation, bilateral PS have a great biomechanical stability and several clinical advantages [2]. Recently, unilateral PS fixation has been suggested as an effective approach as bilateral PS fixation in spinal fusion requiring significantly shorter operating time and hospital stay [3]–[5]. Ding et al. evaluated outcomes of patients with degenerative disc disease following unilateral and bilateral PS fixation procedures respectively and validated the efficacy and safety of unilateral PS fixation in traditional open lumbar interbody fusion. However, this meta-analysis included samples of patients with both one- and two-level degenerative lumbar spine diseases, which might increase the statistical heterogeneity [5]. No similar meta-analyses on unilateral versus bilateral PS fixation in MIS lumbar interbody fusion particularly for one-level degenerative lumbar spine disease are available in the current literature. The objective of this meta-analysis was to examine the effects of unilateral PS fixation and bilateral PS fixation in MIS lumber interbody fusion for one-level degenerative lumbar spine disease by contrasting and combining results from previously reported relevant randomized controlled trials (RCTs) and controlled clinical trials (CCTs).

## Methods

### Literature search

The MEDLINE (through PubMed), EMBASE, Ovid (BIOSIS Previews included), and Cochrane databases were searched through March 18, 2014 using combinations of such key terms as ‘lumbar interbody fusion’, ‘pedicle screw fixation’, ‘minimally invasive’, ‘unilateral’, and ‘bilateral’ with the Boolean operators ‘AND’, ‘NOT’, and ‘OR’. Studies cited in the identified articles were also searched manually. The included studies were published in a peer-reviewed journal as a full article, excluding the gray literature and conference proceedings.

### Inclusion and exclusion criteria

RCTs and CCTs were included if the following criteria were met: (1) they were published in full-text in English in peer-reviewed journals but not in the gray literature and conference proceedings; (2) subjects were diagnosed with one-level lumbar degenerative disease; (3) bilateral and unilateral pedicle screw fixation procedures were comparatively evaluated; and (4) RCTs and comparative observational studies (CCTs). The exclusion criteria were: (1) combined anterior and posterior surgery; (2) lumbar tumors; (3) conditions such as severe osteoporosis, active infection, metabolic disease or symptomatic vascular disease; (4) previous lumbar surgery; and (5) an average follow-up time of <6 months.

### Data extraction

Two reviewers independently screened the titles and abstracts of the retrieved articles to ensure that both inclusion and exclusion criteria were met. Disagreements, if any, were resolved by a third reviewer. Data on the primary outcome measures including visual analogue leg pain score (VAS), Oswestry disability index (ODI), fusion rate and complications and the secondary outcome measures including intra-operative blood loss, operating time and hospital stay were extracted from the included studies and are presented in Table 1.

**Table 1:** Outcome measurements in the studies included in this meta-analysis.

Clinical outcome	Choi Y 2013	Dahdaleh N 2013	Dong J 2014	Lin B 2013	Shen X 2013	Sonmez E 2013
VAS	Yes	Yes	Yes	Yes	Yes	Yes
ODI	Yes	Yes	Yes	Yes	Yes	Yes
JOA	NR	NR	Yes	NR	NR	NR
SF-36	NR	Yes	NR	NR	NR	NR
mProlo scores	NR	NR	NR	NR	Yes	NR
Fusion rate	Yes	Yes	Yes	Yes	Yes	Yes
Complication rate	Yes	Yes	Yes	Yes	Yes	Yes
Operation time	Yes	NR	Yes	Yes	NR	Yes
Blood loss	Yes	Yes	Yes	Yes	NR	Yes
Hospital stay	NR	Yes	Yes	NR	NR	Yes

### Study quality

The methodological quality of the included studies was assessed by two independent authors based on the physiotherapy evidence database (PEDro) scale [6]. This scale is based on a list of 11 criteria, each conferring 1 point to the total score. One criterion was excluded from our calculation of the total PEDro score, and, subsequently there was a potentially maximum total score of 10 points for each of the individual studies included in this meta-analysis. Two reviewers performed the assessment independently. The agreement between the 2 reviewers was evaluated with both the correlation coefficient (r) for interrater agreement and the intraclass correlation coefficient.

### Statistical analysis

The summary statistic was calculated for each individual study using the meta-analysis program of the Cochrane Collaboration (Review Manager 5.2). Binary data (e.g., fusion rate, incidence rate of screw complications) were presented as odds ratio (OR) and 95% confidence intervals (CIs). Continuous outcomes (VAS, ODI, intra-operative blood loss, operative time, and hospital stay) were summarized using the mean difference (MD) and respective 95% CI. Data on the primary and secondary outcome measures were analyzed as continuous or dichotomized variables using random effect model or fixed effect model. When there was no difference between the findings derived with the 2 models, the results were reported using the fixed-effect model, indicating the absence of significant statistical heterogeneity. Random-effects model was used as a more conservative estimate, less likely to reveal a difference between 2 treatment approaches as compared with the fixed-effects model. When any difference between treatment approaches really existed, both models were used. The possibility of publication bias was assessed by funnel plots, showing the intervention effect from each study against the corresponding standard error. Symmetrical and any asymmetry plots would suggest absence and presence of publication bias respectively. The strength and robustness of the pooled results by sequential omission of individual studies were tested by the sensitivity analysis.

## Results

### Eligible studies

**Figure 1:**
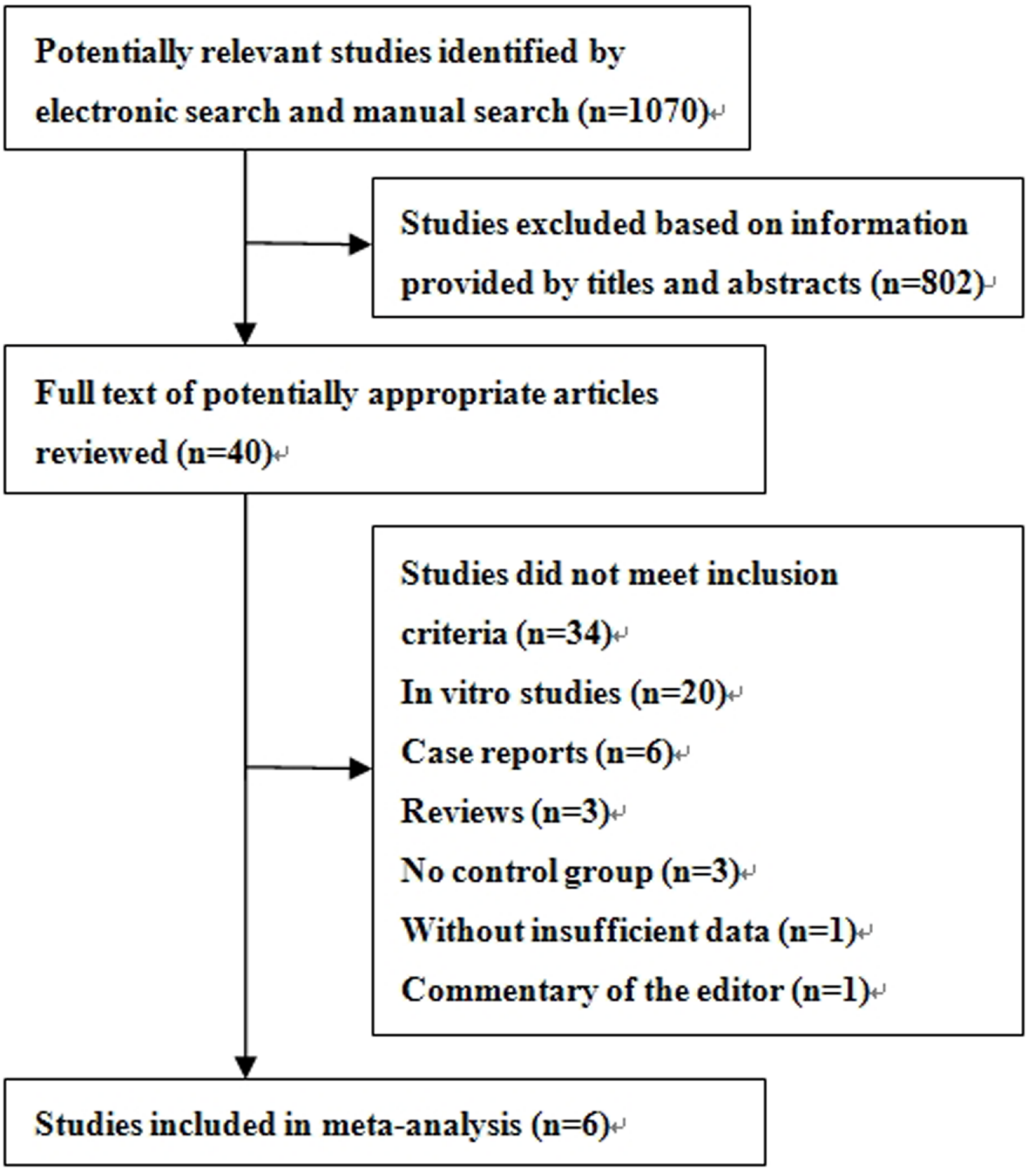
Flow diagram of study identification for this meta-analysis.

A total of 1070 relevant titles were identified through electronic and manual searches. After review of the abstracts and titles, 802 studies were excluded in that they were irrelevant, not on human subjects, not comparative studies or other forms of investigation. Forty articles on bilateral and unilateral PS fixation procedures were retrieved from the databases, and six studies (5 RCTs and 1 CCT) met the inclusion criteria (Figure 1). The characteristics of these six studies [7]–[12] are summarized in Table 2, and no significant differences were found in the baseline characteristics between the two groups. The six studies included a total of 298 participants with 146 adults treated with unilateral PS fixation and 152 adults treated with bilateral unilateral PS fixation.

**Table 2:** Basic characteristics of the studies included in this meta-analysis.

Study	Time	Study design	Etiology	Case	Age	Follow-up	Fusion Technique
Choi Y	2013	RCT	disc herniation, spinal stenosis, degenerativespondylolisthesis, spondylolyticspondylolisthesis, and recurrent disc herniation	U:26	U: 53.39	U: 27.5 months	TLIF
				B:27	B: 56.22	B: 28.9 months	
Dahdaleh N	2013	RCT	single-level degenerative spondylosis orspondylolisthesis, Grade 1 or 2	U: 16	U: 62.2	U: 11.4 months	TLIF
				B: 20	B: 57.3	B: 12.4 months	
Dong J	2014	RCT	single-segment degenerativelumbar instability	U: 20	U: 54.0	U: 36 months	PLIF
				B: 19	B: 56.6	B: 36 months	
Lin B	2013	RCT	spinal canal stenosis,spondylolisthesis(grade I or II), and lumbar disk herniation	U: 43	U: 67	U: 26 months	TLIF
				B: 42	B: 65.5	B: 26 months	
Shen X	2013	RCT	Unilateral lumbar disc herniation,Foraminal stenosis	U: 31	U: 57.3	U: 26.6 months	TLIF
				B: 34	B: 58.9	B: 26.6 months	
Sonmez E	2013	CCT	single level recurrent discherniation	U: 10	U: 47.3	U: 2 year	TLIF
				B: 10	B: 45.6	B: 2 year	

U unilateral fixation, B bilateral fixation.

### Methodological quality

The total scores for all included studies assessed by PEDro quality criteria ranged from 5 to 7 (Table 3). Five studies were considered as being high quality (PEDro score ≥6), and one study was of low quality (PEDro score <6). The two independent reviewers reached consensuses on the scoring of all items without any disagreement.

**Table 3:** Methodological quality of the included studies assessed with the PEDro scale.

Study	Item PEDro score										Total score
	2	3	4	5	6	7	8	9	10	11	
Choi Y	+	−	+	−	−	+	+	+	+	+	7/10
Dahdaleh N	+	−	+	−	−	−	+	+	+	+	6/10
Dong J	+	−	+	−	−	−	+	+	+	+	6/10
Lin B	+	−	+	−	−	−	+	+	+	+	6/10
Shen X	+	−	+	−	−	−	+	+	+	+	6/10
Sonmez E	−	−	+	−	−	−	+	+	+	+	5/10

### Postoperative functional performance (VAS and ODI)

**Figure 2:**
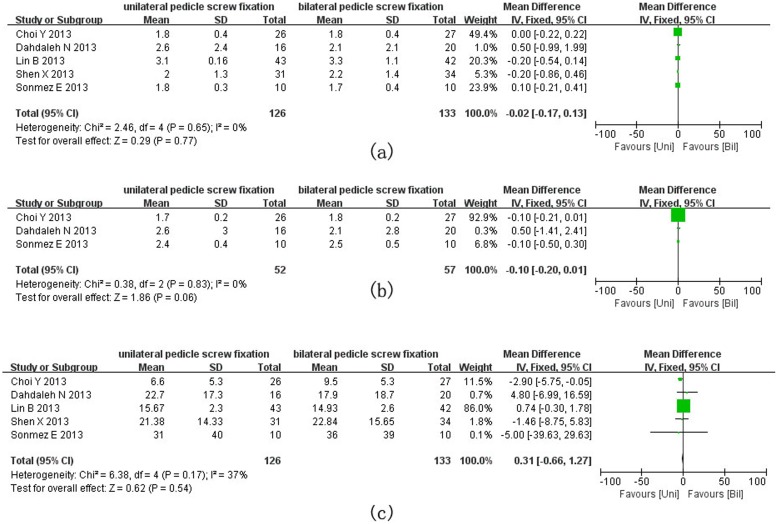
Forest plots for VAS for back pain (a), VAS for leg pain (b) and ODI (c) in patients undergoing unilateral and bilateral PS fixations respectively.

VAS and ODI are the most frequently used variables to assess the postoperative function performance of patients. Five studies assessed VAS for back pain, which was not significantly different between bilateral and unilateral groups (MD = −0.02, 95% CI = −0.17 to 0.13; P = 0.77) (Figure 2a). Three studies assessed VAS for leg pain, which was not different between the two groups either (MD = −0.10, 95% CI = −0.20 to 0.01; P = 0.06) (Figure 2b). ODI scores were available in five trials [16], [19]–[22] where no significant difference was detected between the two groups (MD = 0.31, 95% CI = −0.66 to 1.27; P = 0.54) (Figure 2c).

### Fusion rate and complications

**Figure 3:**
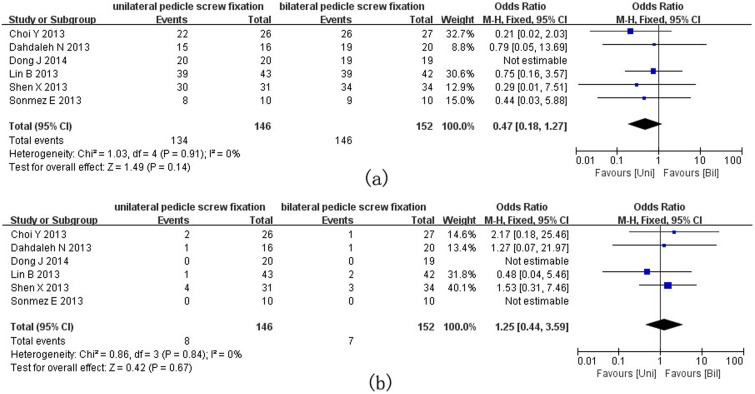
Forest plots for fusion rate (a) and incidence rate of complications (b) in patients undergoing unilateral and bilateral PS fixations respectively.

The overall fusion rate was 91.8% (134/146) in the unilateral group and 96.0% (146/152) in the bilateral group (OR = 0.47, 95% CI = 0.18–1.27, P = 0.14, Figure 3a). No nonunion case was reported in the study of Dong [9]. The overall incidence of complications in the unilateral and bilateral groups was 5.48% (8/146) and 4.61% (7/152), respectively, and the difference was not significant (OR = 1.25, 95% CI = 0.44–3.59, P = 0.67, Figure 3b). No complications were observed in two studies [8], [9].

### Operation time, blood loss and hospital stay

**Figure 4:**
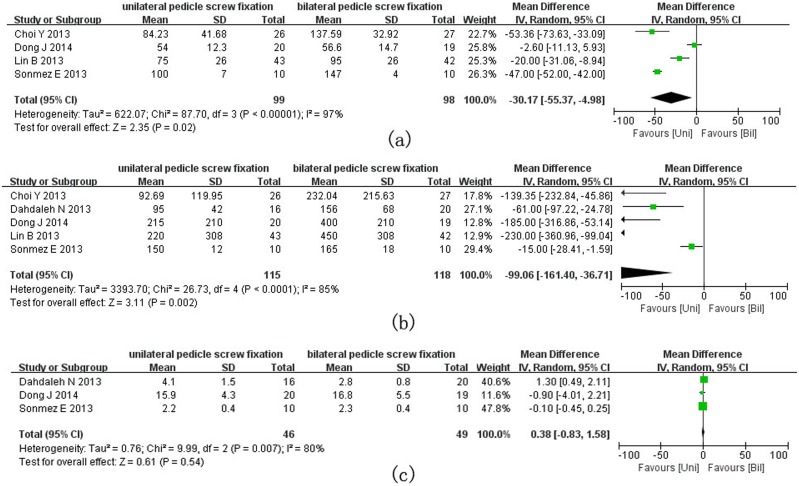
Forest plots for operation time (a), blood loss (b) and hospital stay (c) in patients undergoing unilateral and bilateral PS fixations respectively.

Operation time was assessed in four eligible studies and the results of the meta-analysis are presented in Figure 4a. Overall, unilateral PS fixation required a significantly less operative time as compared with bilateral PS fixation (MD = −30.17, 95% CI = −55.37 to −4.98; P = 0.02). Shown as a forest plot in Figure 4b are the results of meta-analysis of intra-operative blood loss; the amount of blood loss was significantly smaller in the unilateral group than in the bilateral group (MD = −99.06, 95% CI = −161.40 to −36.71; P = 0.002). Three studies reported the mean length of hospital stay where no significant difference was detected between the unilateral and bilateral groups (MD = 0.38, 95% CI = −0.83 to 1.58; P = 0.54).

## Discussion

Bilateral PS fixation after lumbar interbody fusion is accepted as a standard procedure. Providing rigid fixation, bilateral PS fixation has a great biomechanical stability and clinical benefits. However, the rigidity of bilateral PS fixation can lead to device-related osteoporosis of the vertebrae [13] and makes the adjacent segment prone to load- and motion-induced degeneration [14]. To achieve optimal biomechanical conditions in the fused segment and minimize adverse effects in the adjacent levels caused by instrumentation, the use of less rigid systems of fixation has been advocated [15]. Some recent clinical and biomechanical studies on the suitability of unilateral PS fixation have demonstrated that a reliable fusion with fewer pedicle screws can be achieved [16], [17]. Nevertheless, unilateral PS fixation may be detrimental to spine stability and the promotion for fusion as suggested by an *in vitro* study [18]. Therefore, the use of unilateral or bilateral PS fixation remains a matter of debate.

Numerous previous biomechanical and clinical studies attempted to comparatively evaluate unilateral and bilateral PS fixation approach and inconsistent results were obtained. Chen et al. [16] demonstrated that unilateral PS fixation was good enough to maintain the stability of the spine in a biomechanics study. Goel et al. [19] reported that the unilateral PS system was effective to reduce stress shielding of the vertebra and diminish peak stress arising in the adjacent levels above and below the fusion. Toyone and coauthors [20] recently reported that unilateral PS fixation was associated with a low incidence of adjacent-segment degeneration following posterior lumber interbody fusion. However, an increasing number of published studies have raised concerns over the clinical benefits of unilateral fixation. Yucesoy et al. [21] reported that unilateral PS fixation was inadequate to stabilize a 2-level unilateral lesion when compared with bilateral fixation. Aoki et al. [22] observed that unilateral fixation caused postoperative cage migration more frequently than bilateral fixation in patients who had scoliotic curvature with a Cobb angle >10°. In addition, Slucky et al. showed that unilateral PS fixation supplied only half of the improvement in stiffness compared bilateral PS fixation and caused significant off-axis rotational motions, which could hinder stability and the promotion for fusion after transforaminal lumbar interbody fusion (TLIF) [18].

However, unilateral PS fixation supplemented with translaminar facet screw fixation on the contralateral side offered stability comparable to that offered by bilateral PS fixation. Several other biomechanical and clinical studies also showed that supplementation of a contralateral facet screw might exert a similar effect as bilateral PS fixation on the stiffness or range of motion following TLIF [23]–[25]. Therefore supplementation of a contralateral facet screw may possibly compensate the limitations of unilateral PS fixation in lumbar interbody fusion.

Spinal fusion can be achieved by both posterolateral and/or interbody fusion techniques [26]. However, conventional spinal fusion is related to significant muscle stripping and retraction that can adversely affect both short- and long-term patient outcomes [1]. In contrast, minimally invasive spinal fusion is performed by a muscle-dilating approach, which may significantly minimize or diminish the iatrogenic soft tissue injury, intra-operative blood loss, postoperative pain and the duration of hospital stays [27]. Accordingly, many surgeons prefer minimally invasive methods, such as TLIF with unilateral PS fixation [1], [4], [28], and believe that unilateral PS fixation is sufficient to accomplish spinal fusion. The objective of this meta-analysis was to systematically compare the efficiency and safety of unilateral and bilateral PS fixation procedures in MIS lumbar interbody fusion for one-level lumbar degenerative disease.

Our analysis suggested that there were no differences between unilateral PS fixation and bilateral PS fixation in VAS and ODI. This finding was in agreement with the results from some previous studies [9]–[11] where the patient outcomes were evaluated either using other assessment systems such as the Japanese Orthopaedic Association (JOA), 36-Item Short Form Healthy Survey version 2 (SF-36v2) and mProlo scores respectively or using radiographic parameters such as the whole lumbar lordosis, the segmental lordosis, fusion level disc space angle, lumbar scoliosis angle, and segmental scoliosis angle [11].

There were significantly less blood loss and significantly shorter operation time in the unilateral PS fixation group as compared with the bilateral PS fixation group in our meta-analysis. Unilateral PS fixation dissects soft tissue and insert pedicle screws only on one side and therefore it takes less time and decreases blood loss. Moreover, less soft tissue dissection may allow for early recovery [28]. However, the average length of hospital stay was similar in the two groups in our meta-analysis, which was inconsistent with the observation of a previous study where the hospital stay was shorter for unilateral fixation than for bilateral fixation due to early recovery and rehabilitation [7]. One of the reasons for the discrepancy might be the small number of studies included in our meta-analysis. Another reason might be the high heterogeneity among the included studies; in one study the hospital stay was longer in the unilateral group than in the bilateral group because of pulmonary edema in some patients.

Despite no statistical difference, the overall fusion rate was slightly lower in the unilateral group (91.8%) than in the bilateral group (96%). It was likely that less biomechanical stability in unilateral instrumentation might have negatively impacted the fusion. Because of this, supplementation of a contralateral facet screw has been proposed as a solution to compensate for the insufficient stability of unilateral PS fixation. The incidences of complications in unilateral and bilateral groups were 5.48% (8/146) and 4.61% (7/152), respectively. No single complication was identified in two studies. This finding was inconsistent with results from many previous studies where insufficient stability of unilateral PS fixation increased the incidence of cage migration [12], [22].

Our study has a number of weaknesses. First of all, in some of the included studies, there were methodological limitations including failure to collect data prospectively, nonconsecutive enrollment of patients, inadequate baseline comparisons, and improper blinding or non-blinding. And these limitations somewhat reduced the level of evidence for this meta-analysis. Secondly, heterogeneity existed among the studies particularly when the continuous outcome measure variables were pooled. The heterogeneity might be attributable to differences among the included studies in the study design, study quality, patients’ characteristics, and the diverse technical specifications. Thirdly, multiple assessment tools and fusion criteria were used in the included studies that might have confounded the combined results. Lastly, incomplete data recording was observed in some of the included studies. Pooling of such data might lead to bias.

In summary, through contrasting and combining results from 5 RCTs and 1 CCT on unilateral versus bilateral PS fixation in MIS lumbar fusion, this meta-analysis has shown that unilateral PS fixation may significantly reduce the intraoperative blood loss and shorten the operation time, somewhat improve the clinical outcome scores of ODI and VAS and fusion rate, and significantly decrease the incidence rate of complications while not affecting hospital stay as compared with bilateral PS fixation in patients with one-level lumbar degenerative disease. These findings suggest that unilateral PS fixation in MIS lumbar fusion is as effective and safe as but less time-consuming than bilateral PS fixation for one level lumbar degenerative disease. Nevertheless, this approach warrants further evaluations in high quality RCTs with large sample size and long-term follow-up before its wider clinical application.

## Supporting Information

Checklist S1 **PRISMA Checklist.**
(DOC)
